# The Predictive Score for Patients Hospitalized With COVID-19 in Resource-Limited Settings

**DOI:** 10.7759/cureus.30373

**Published:** 2022-10-17

**Authors:** Chepsy Philip, Alice David, S K Mathew, Sanjo Sunny, Vijaya Kumar K, Linda Jacob, Luke Mathew, Suresh Kumar, George Chandy

**Affiliations:** 1 Clinical Hematology and Bone Marrow Transplant, COVID-19 Research Group, Believers Church Medical College Hospital, Thiruvalla, IND; 2 Medical Research, COVID-19 Research Group, Believers Church Medical College Hospital, Thiruvalla, IND; 3 Internal Medicine, Believers Church Medical College Hospital, Thiruvalla, IND; 4 Intensive Care Unit, Believers Church Medical College Hospital, Thiruvalla, IND; 5 Pharmacology and Therapeutics, COVID-19 Registry Group, Believers Church Medical College Hospital, Thiruvalla, IND; 6 Pulmonary Medicine, COVID-19 Registry Group, Believers Church Medical College Hospital, Thiruvalla, IND; 7 Pediatric Cardiology, COVID-19 Research Group, Believers Church Medical College Hospital, Thiruvalla, IND; 8 Gastroenterology and Hepatology, Believers Church Medical College Hospital, Thiruvalla, IND

**Keywords:** covid-19 retro, risk calculators, disease mortality, icu admissions, scores, covid-19

## Abstract

Background and aims

The second wave of coronavirus disease 2019 (COVID-19) has been devastating in India and many developing countries. The mortality reported has been 40% higher than in the first wave, overwhelming the nation’s health infrastructure. Despite a better understanding of the disease and established treatment protocols including steroids and heparin, the second wave was disastrous. Subsequent waves have the potential to further cripple healthcare deliveries, also affecting non-COVID-19 care across many developing economies. It is then important to identify and triage high-risk patients to best use the limited resources. Routine tests such as neutrophil and monocyte counts have been identified but have not been successfully validated uniformly, and their utility is still being understood in COVID-19.

Various predictive models that are available require online resources and calculators and additionally await validation across all populations. These, although useful, might not be available or accessible across all institutions. It is then important to identify easy-to-use scores that utilize tests done routinely.

In identifying with this goal, we did a retrospective review of the institutional database to identify potential predictors of intensive care unit (ICU) admission and mortality in patients hospitalized during the second wave who accessed healthcare at our academic setup.

Results

Three predictors of mortality and four predictors of ICU admission were identified. Absolute neutrophil count was a common predictor of both ICU admission and mortality but with two separate cut points. An absolute neutrophil count of >4,200 predicted need for ICU admission (odds ratio (OR): 3.1 (95% confidence interval (CI): 2.0, 4.8)), and >7,200 predicted mortality (adjusted OR: 4.2 (95% CI: 1.9, 9.4)). We observed that a blood urea level greater than 45 was predictive of needing ICU care (adjusted OR: 8.0 (95% CI: 3.7, 17.6)). In our dataset, serum ferritin of >500 was predictive of ICU admission (adjusted OR: 2.7 (95% CI: 1.2, 5.9)). We noted a right shift of partial pressure (p50 is the oxygen tension at which hemoglobin is 50% saturated) (p50c) in SARS-CoV-2 as a predictor of ICU care (OR: 2.6 (95% CI: 1.7, 3.9)) when partial pressure is >26.5. In our analysis, a serum protein of less than 7 g/dL (OR: 2.8 (95% CI: 1.7, 4.4)) was a predictive variable for ICU admission. An LDH value of >675 was predictive of severity with a need for ICU admission (OR: 9.2 (95% CI: 5.4, 15.5)) in our series. We then assigned a score to each of the predictive variables based on the adjusted odds ratio.

Conclusion

We identified a set of easy-to-use predictive variables and scores to recognize the subset of patients hospitalized with COVID-19 with the highest risk of death or clinical worsening requiring ICU care.

## Introduction

Pandemics and outbreaks affect resources in multiple ways, making their impact difficult to predict [1]. The national data on coronavirus disease 2019 (COVID-19) in India is 478,759 as of December 23, 2021 [2]. Although the case fatality rate for COVID-19 is lower than that observed in high-income countries, the second wave of COVID-19 has been devastating in India and many developing countries [3].

The number of infected people and casualties in the current COVID-19 pandemic is evidence that despite improved understanding, better protocols, and attempts to plan, the global healthcare systems remain unprepared. Subsequent waves and strains can cripple further healthcare deliveries, affecting non-COVID-19 care across many developing economies. It is then essential to identify and triage high-risk patients to best use the limited resources. Various predictive models are available that are not yet validated across all populations and require online resources and calculators [4]. These, although helpful, might not be available or accessible across all institutions. Therefore, it is vital to identify easy-to-use scores that utilize tests done routinely.

The many different challenges for treatment in resource-limited settings, including delayed presentation, higher disease burden, and poor general condition, require resource-specific solutions. We attempted to develop a multivariate model of the COVID-19 severity score using baseline investigations relevant to our clinical setting in an attempt to identify the subset of patients with the highest risk of death or clinical worsening requiring intensive care unit (ICU) care.

Specifically, the objectives were to identify potential predictors of mortality and potential predictors of ICU admission in patients admitted with COVID-19.

## Materials and methods

Data collection

The ethics committee of the Believers Church Medical College Hospital approved this study. Written informed consent was waived owing to the use of de-identified retrospective data. On behalf of the institutional COVID-19 research group, we established a retrospective cohort to study COVID-19 cases in the second wave at our institute. We obtained medical records from laboratory-confirmed hospitalized cases with COVID-19 between February 1 and June 15, 2021.

COVID-19 diagnoses were confirmed by positive real-time reverse-transcription polymerase chain reaction (RT-PCR) assay or antigen testing using nasal and pharyngeal swab specimens. A team of experienced clinicians and clinical specialists reviewed, abstracted, and cross-checked the data. We included all patients with data on clinical status at hospitalization (laboratory findings, clinical symptoms and signs, severity, and discharge status).

Potential predictor variables

Potential predictive variables included the following patient characteristics at hospital admission: sociodemographic variables, laboratory findings, and medical history. Sociodemographic variables collected for the study included age, gender, area of residence, and occupation. The occupation was recorded as COVID-19 warrior if they were doctors, nurses, medical cleaners, pathologists, paramedics, ambulance drivers, or healthcare administrators working in facilities attending to patients with COVID-19. Medical history included several comorbidities (pulmonary, cardiac, renal hepatic, and vascular). Laboratory findings included arterial blood gas (ABG) analytics (partial arterial oxygen pressure and oxygen saturation), hematologic parameters (white blood cell, lymphocyte, platelet counts, neutrophil count, and hemoglobin levels), markers of inflammation (C-reactive protein (CRP), erythrocyte sedimentation rate (ESR), and ferritin), coagulation parameters (D-dimer levels, prothrombin time, and activated partial thromboplastin time), and metabolic panel (liver function tests, renal function tests, procalcitonin, lactate dehydrogenase (LDH), serum sodium, serum potassium, serum chlorine, and blood glucose).

Outcomes

Patient Outcomes

We defined the endpoints as admission to ICU and death. We chose these endpoints because admission to ICU and death are severe outcomes of COVID-19 that have been adopted in previous studies to assess the severity of other serious infectious diseases. The outcome was determined by reviewing the patient’s records.

Statistical methods

Descriptive statistics included mean and standard deviation (SD) for continuous variables and frequencies and percentages for categorical variables.

Univariate analysis of all potential confounders and other risk factors was done using Student’s t-test after log-transforming non-normal variables or the χ2 test for categorical variables. Multicollinearity was assessed by first grouping all laboratory parameters into clinically related subsets (ABG, hemogram, markers of inflammation, thrombotic markers, renal function, liver function, metabolic markers, and markers of blood glucose) and then conducting principal component analysis. The final components of each cluster were picked by two authors when the lowest 1-R square ratios were similar. The final model was based on the logistic regression model using the backward elimination method. The discriminatory power of each significant variable of the final model was assessed using the area under the curve (AUC) of a receiver operating characteristic (ROC) curve. These variables were converted to binary using cutoffs based on Youden’s J index and a minimum sensitivity and specificity of 50%. The validity of these cut points was assessed using sensitivity, specificity, positive and negative predictive values, and positive likelihood ratio (LR+) in this development cohort. The beta coefficient of the logistic regression model containing these binary variables was taken as the score to measure the severity of COVID-19.

## Results

The study cohort comprised 757 subjects whose mean age was 56.0 ± 19.0 years. There were 426 (56.3%) males. Of the subjects, 713 (94.2%) were from the three districts (Pathanamthitta, Alappuzha, and Kottayam) we serve, and eight (1.1%) were COVID-19 warriors. A total of 136 (18%) patients in the study cohort needed ICU admission, and 78 (10.3%) patients died.

Predictor selection

Variable Selection and Score Construction: Mortality Predictors

We started with 57 total variables, which was reduced to 30 after univariate analysis and was trimmed to 17 after removing highly correlated variables. The final model included only three significant variables: count (absolute neutrophil), urea, and ferritin (CUF) (Table 1).

**Table 1:** Potential predictors of mortality among patients hospitalized with COVID-19 ICU: intensive care unit; N: number; SD: standard deviation; ABG: arterial blood gas; CCA2: calcium; CCL: chloride; CHCO3: bicarbonate; FHHb: hemoglobin spectrophotometry; PCO2: partial pressure of CO2; PO2: partial pressure of O2; SO2: oxygen saturation; cLac: lactate; ctCO2BC: carbon dioxide concentration; ctO2c: oxygen concentration; p50c: p50 (p50 is the oxygen tension at which hemoglobin is 50% saturated) value of a blood gas sample; pO2aAe: oxygen tension (based indices of oxygenation); FShuntE: measurement and estimation of shunt; CRP: C-reactive protein; FER: ferritin; ESR: erythrocyte sedimentation rate; CGLU: glucose estimated with ABG; PTT1: prothrombin time; PTTACT: activated partial thrombin time; AGRatio: albumin/globulin ratio; ALTSGPT: alanine transaminase; ASTSGOT: aspartate transaminase; AlkalinePh: alkaline phosphatase; BiLDIR: direct bilirubin; BilIndir: indirect bilirubin; BilTOT: total bilirubin; LACTATEDH: lactate dehydrogenase (LDH); Hb: hemoglobin; MCV: mean corpuscular volume; PCV: packed cell volume; Retic: reticulocyte; LHD: low hemoglobin density; MSCV: mean sphered cell volume; RSF: red blood cell size factor; EosCNT: eosinophil count; LymCNT: lymphocyte count; MonoCNT: monocyte count; PolyCNT: polymorph count; TLC: total leucocyte count; INR: international normalized ratio; HbA1C: hemoglobin A1C

Variable	ICU		Ward		P value
	N	Mean ± SD/N (%)	N	Mean ± SD/N (%)	
Age	136	62.1 ± 16.0	621	54.6 ± 19.3	<0.0001
Gender (male)	136	84 (69.1%)	621	331 (53%)	0.0008
Occupation (COVID-19 warriors)	103	0 (0%)	617	8 (1.3%)	0.2452
Residence	136	129 (94.9%)	621	586 (94.4%)	0.82
ABG					
Anion	115	7.7 ± 3.8	429	7.57 ± 4.1	0.17
CCA2	114	1.2 ± 0.1	428	1.16 ± 0.1	0.50
CCL	115	105.6 ± 5.4	429	105.0 ± 4.9	0.30
CHCO3	115	21.1 ± 4.2	429	22.4 ± 3.2	0.002
FHHb	115	14.7 ± 15.2	429	10.2 ± 13.0	<0.0001
PCO2	115	33.4 ± 10.1	429	34.5 ± 7.1	0.06
PH	115	7.4 ± 0.1	429	7.4 ± 0.1	0.18
PO2	115	66.3 ± 39.8	429	69.9 ± 20.7	0.01
SO2	115	85.0 ± 15.4	429	89.6 ± 13.2	0.02
cLac	115	1.8 ± 1.2	429	1.4 ± 0.7	<0.0001
ctCO2BC	115	41.7 ± 8.4	429	44.2 ± 6.5	0.003
ctO2c	114	16.5 ± 3.7	429	17.0 ± 3.6	0.19
p50c	115	27.1 ± 3.0	429	25.8 ± 2.4	<0.0001
pO2aAe	114	61.8 ± 44.8	429	63.8 ± 19.4	0.01
FShuntE	111	27.6 ± 19.0	428	18.9 ± 17.9	<0.0001
Hemogram					
Hb	131	13.0 ± 2.1	577	13.1 ± 1.9	0.41
MCV	131	86.3 ± 5.8	577	85.9 ± 6.1	0.54
PCV	131	38.7 ± 5.7	577	39.2 ± 5.6	0.31
Platelets	131	2.2 ± 0.9	573	2.2 ± 0.8	0.27
Retic	131	1.1 ± 0.7	574	1.1 ± 0.7	0.85
LHD	72	5.6 ± 9.8	309	5.0 ± 7.6	0.49
MSCV	71	80.8 ± 6.8	306	78.5 ± 5.9	0.01
RSF	71	94.1 ± 6.9	306	92.9 ± 5.8	0.18
EosCNT	129	80.0 ± 135.6	571	94.6 ± 196.7	0.31
LymCNT	129	1,357.6 ± 882.6	571	1,680.8 ± 911.3	<0.0001
MonoCNT	129	200.0 ± 145.6	571	222.3 ± 145.20	0.91
PolyCNT	130	7,355.3 ± 4,772.4	571	5,007.9 ± 2,983.4	<0.0001
TLC	131	9,034.1 ± 5,094.5	576	6,983.2 ± 3,196.0	<0.0001
Renal function					
LACTATEDH	85	945.3 ± 498.9	398	555.4 ± 214.1	<0.0001
Potassium	126	4.3 ± 0.8	534	4.09 ± 0.5	0.01
Protein	98	6.8 ± 0.6	416	7.09 ± 0.5	<0.0001
Sodium	127	133.4 ± 5.5	546	134.7 ± 5.0	0.01
Urea	121	59.2 ± 50.9	506	33.97 ± 33.1	<0.0001
Creatinine	126	1.9 ± 2.5	563	1.2 ± 1.9	<0.0001
Liver function					
AGRatio	98	1.1 ± 0.2	416	1.3 ± 0.2	<0.0001
ALTSGPT	98	51.6 ± 68.2	416	44.7 ± 45.4	0.16
ASTSGOT	98	69.5 ± 94.2	416	47.1 ± 35.1	0.001
Albumin	98	3.6 ± 0.4	421	3.9 ± 0.5	<0.0001
AlkalinePh	98	76.8 ± 40.4	416	74.5 ± 38.8	0.67
BiLDIR	98	0.3 ± 0.4	416	0.2 ± 0.2	<0.0001
BilIndir	98	0.6 ± 0.4	416	0.5 ± 0.4	0.006
BilTOT	98	0.8 ± 0.7	416	0.7 ± 0.5	<0.0001
Globulin	98	3.2 ± 0.4	416	3.2 ± 0.4	0.41
Thrombotic markers					
INR	46	1.1 ± 0.3	68	1.09 ± 0.32	0.99
D-dimer	105	2,081.7 ± 4,458.6	482	915.9 ± 1,569.5	<0.0001
PTT1	46	14.3 ± 3.5	67	14.3 ± 3.6	0.97
PTTACT	36	31.6 ± 6.5	33	31.0 ± 5.9	0.64
Blood glucose markers					
CGLU	115	225.2 ± 120.9	429	181.0 ± 90.7	<0.0001
Glucose	36	195.1 ± 63.1	123	174.5 ± 66.2	0.049
HbA1C	36	8.4 ± 2.2	123	7.7 ± 2.3	0.06
Inflammation markers					
CRP	110	78.9 ± 73.4	536	39.1 ± 5 4.3	<0.0001
FER	76	970.5 ± 889.6	389	426.0 ± 539.5	<0.0001
ESR	10	49.5 ± 28.5	38	27.9 ± 23.5	0.01

**Figure 1:**
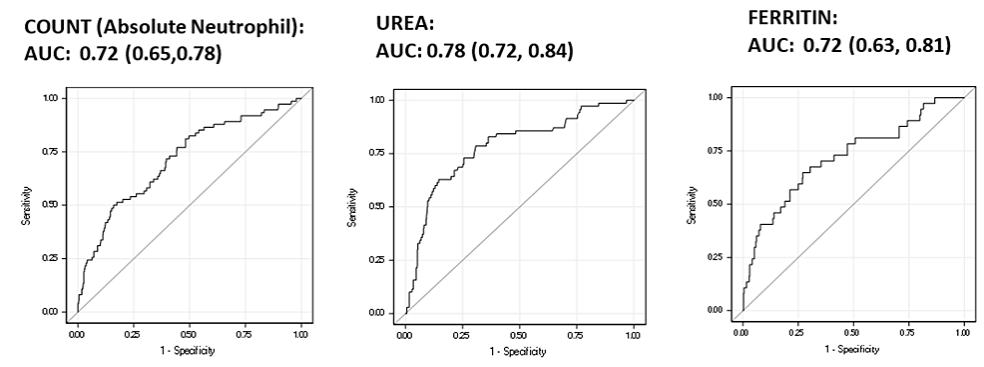
ROC curves depicting the power to discriminate by mortality ROC: receiver operating characteristic; AUC: area under the curve

The area under the curve (AUC) for all three predictors of mortality is clearly above 0.50, and as shown in Figure 1, the curves do not cross the line of no discrimination at any point. The AUC (95% confidence interval (CI)) is 0.72 (0.65, 0.78) for the absolute neutrophil count, 0.78 (0.72, 0.84) for urea, and 0.72 (0.63, 0.81) for ferritin. With these three predictors included in a single model, the AUC (95% CI) was 0.77 (0.71, 0.83).

The cut point to discriminate those with a higher risk of mortality based on Youden’s J index was as follows: >7,200 for the absolute neutrophil count, >45 for urea, and >500 for ferritin. A minimum value of 0.50 was ensured for sensitivity and specificity while determining the cut point (Table 2).

**Table 2:** Measures of validity of predictors of mortality

Mortality	Count (absolute neutrophil)	Urea	Ferritin
Cut point	>7,200	>45	>500
Sensitivity	0.50 (0.38, 0.62)	0.63 (0.50, 0.74)	0.62 (0.45, 0.78)
Specificity	0.84 (0.81, 0.87)	0.85 (0.82, 0.88)	0.73 (0.69, 0.77)
LR+	3.13 (2.00, 4.70)	4.27 (2.81, 6.24)	2.31 (1.43, 3.41)

The sensitivity and specificity of each of the three predictors are as follows: 0.50 (0.38, 0.62) and 0.84 (0.81, 0.87) for the absolute neutrophil count, 0.63 (0.50, 0.74) and 0.85 (0.82, 0.88) for urea, and 0.62 (0.45, 0.7) and 0.73 (0.69, 0.7) for ferritin, respectively. The positive likelihood ratio (LR+) of each of the three predictors is as follows: 3.13 (2.00, 4.70) for the absolute neutrophil count, 4.27 (2.81, 6.24) for urea, and 2.31 (1.43, 3.41) for ferritin.

As shown in Table 3, the allocation of points is as follows: 4 points for the absolute neutrophil count, eight points for urea, and three points for ferritin. For all other values, the points allotted are zero. With a score of 7 as a cut point, the sensitivity and specificity are 0.63 (0.51, 0.74) and 0.86 (0.83, 0.89), respectively, and the positive likelihood ratio is 4.54 (3.07, 6.49). The risk of mortality is 10.5 (6.3, 17.5) times higher if the total score is greater than 7, showing that any two of these three factors synergistically affect death when present together.

**Table 3:** Allocation of points for predictors of mortality OR: odds ratio; CI: confidence interval

Predictors	Cut point	OR (95% CI)	Adjusted OR (95% CI)	Score
Absolute neutrophil count	>7,200	5.3 (3.2, 8.7)	4.2 (1.9, 9.4)	4
Urea	>45	9.8 (5.7, 16.8)	8.0 (3.7, 17.6)	8
Ferritin	>500	4.5 (2.2, 8.9)	2.7 (1.2, 5.9)	3

Variable Selection and Score Construction: ICU Predictors

We started with 57 total variables, which was reduced to 33 after univariate analysis and further trimmed to 19 after removing highly correlated variables. The final model included only four significant variables: absolute neutrophil count, p50c (partial pressure/p50 is the oxygen tension at which hemoglobin is 50% saturated), protein, and LDH (APPL) (Table 4).

**Table 4:** Potential predictors for ICU admission among patients hospitalized with COVID-19 ICU: intensive care unit; N: number; SD: standard deviation; ABG: arterial blood gas; CCA2: calcium; CCL: chloride; CHCO3: bicarbonate; FHHb: hemoglobin spectrophotometry; PCO2: partial pressure of CO2; PO2: partial pressure of O2; SO2: oxygen saturation; cLac: lactate; ctCO2BC: carbon dioxide concentration; ctO2c: oxygen concentration; p50c: p50 (p50 is the oxygen tension at which hemoglobin is 50% saturated) value of a blood gas sample; pO2aAe: oxygen tension (based indices of oxygenation); FShuntE: measurement and estimation of shunt; CRP: C-reactive protein; FER: ferritin; ESR: erythrocyte sedimentation rate; CGLU: glucose estimated with ABG; PTT1: prothrombin time; PTTACT: activated partial thrombin time; AGRatio: albumin/globulin ratio; ALTSGPT: alanine transaminase; ASTSGOT: aspartate transaminase; AlkalinePh: alkaline phosphatase; BiLDIR: direct bilirubin; BilIndir: indirect bilirubin; BilTOT: total bilirubin; LACTATEDH: lactate dehydrogenase (LDH); Hb: hemoglobin; MCV: mean corpuscular volume; PCV: packed cell volume; Retic: reticulocyte; LHD: low hemoglobin density; MSCV: mean sphered cell volume; RSF: red blood cell size factor; EosCNT: eosinophil count; LymCNT: lymphocyte count; MonoCNT: monocyte count; PolyCNT: polymorph count; TLC: total leucocyte count; INR: international normalized ratio; HbA1C: hemoglobin A1C

Variables	Dead		Alive		P value
	N	Mean ± SD	N	Mean ± SD	
Age	78	66.7 ± 12.4	679	54.7 ± 19.2	<0.0001
Sex (male)	78	38 (48.7%)	679	387 (57%)	0.16
Occupation (COVID-19 warriors)	64	0 (0%)	656	8 (1.2%)	0.37
Residence (catchment)	78	73 (93.6%)	659	642 (94.6%)	0.73
ABG					
Anion	65	8.8 ± 4.4	479	7.4 ± 3.9	0.04
CCA2	65	1.2 ± 0.1	477	1.2 ± 0.1	0.88
CCL	65	104.9 ± 5.4	479	105.2 ± 4.9	0.73
CHCO3	65	20.8 ± 4.5	479	22.3 ± 3.3	0.02
FHHb	65	15.6 ± 16.8	479	10.6 ± 13.0	0.001
PCO2	65	33.3 ± 11.2	479	34.4 ± 7.2	0.12
PH	65	7.4 ± 0.1	479	7.4 ± 0.1	0.28
PO2	65	65.5 ± 44.2	479	69.6 ± 22.4	0.03
SO2	65	84.2 ± 17.0	479	89.3 ± 13.2	0.06
cLac	65	1.8 ± 0.9	479	1.4 ± 0.8	0.001
ctCO2BC	65	41.3 ± 9.1	479	44.0 ± 6.6	0.02
ctO2c	65	15.9 ± 4.0	478	17.0 ± 3.6	0.07
p50c	65	27.2 ± 3.3	479	25.9 ± 2.4	0.003
pO2aAe	65	61.5 ± 53.5	478	63.6 ± 20.7	0.03
FShuntE	63	29.5 ± 19.3	476	19.5 ± 18.0	<0.0001
Hemogram					
Hb	74	12.7 ± 2.4	634	13.1 ± 1.9	0.12
MCV	74	86.3 ± 6.4	634	86.0 ± 6.0	0.68
PCV	74	38.1 ± 6.7	634	39.3 ± 5.4	0.10
Platelets	74	2.2 ± 1.1	630	2.2 ± 0.8	0.23
Retic	74	1.2 ± 0.7	631	1.1 ± 0.7	0.02
LHD	44	6.0 ± 10.2	337	5.0 ± 7.7	0.69
MSCV	43	81.4 ± 7.3	334	78.6 ± 5.9	0.01
RSF	43	94.7 ± 7.6	334	92.9 ± 5.8	0.18
EosCNT	73	92.5 ± 169.1	627	91.8 ± 189.0	0.03
LymCNT	73	1,410.7 ± 1,056.6	627	1,645.7 ± 893.8	0.002
MonoCNT	73	212.3 ± 167.9	627	218.9 ± 142.7	0.04
PolyCNT	74	8,403.4 ± 5,611.0	627	5,093.8 ± 2,983.4	<0.0001
TLC	74	10,127.3 ± 6,024.3	633	7,040.1 ± 3,186.0	<0.0001
Renal function					
LACTATEDH	43	997.0 ± 507.3	440	587.5 ± 271.6	<0.0001
Potassium	70	4.4 ± 0.9	590	4.1 ± 0.6	0.04
Protein	51	6.8 ± 0.6	463	7.1 ± 0.5	0.002
Sodium	71	133.4 ± 5.4	602	134.6 ± 5.1	0.07
Urea	70	70.2 ± 52.9	557	34.9 ± 34.3	<0.0001
Creatinine	73	2.4 ± 2.9	616	1.2 ± 1.8	<0.0001
Liver function					
AGRatio	51	1.1 ± 0.2	463	1.2 ± 0.2	<0.0001
ALTSGPT	51	43.9 ± 34.9	463	46.2 ± 52.0	0.51
ASTSGOT	51	57.8 ± 41.1	463	50.6 ± 53.5	0.07
Albumin	51	3.5 ± 0.5	468	3.9 ± 0.5	<0.0001
AlkalinePh	51	76.5 ± 42.3	463	74.8 ± 38.7	0.78
BiLDIR	51	0.2 ± 0.3	463	0.2 ± 0.2	<0.0001
BilIndir	51	0.6 ± 0.2	463	0.5 ± 0.4	0.03
BilTOT	51	0.8 ± 0.5	463	0.7 ± 0.6	0.01
Globulin	51	3.3 ± 0.4	463	3.2 ± 0.4	0.06
Thrombotic markers					
INR	24	1.3 ± 0.6	90	1.1 ± 0.2	0.08
DDIMER	55	1,709.6 ± 2,384.8	532	1,063.9 ± 2,393.3	0.003
PTT1	24	16.1 ± 6.0	89	13.9 ± 2.3	0.08
PTTACT	19	31.4 ± 7.3	50	31.3 ± 5.8	0.93
Blood glucose markers					
CGLU	65	230.1 ± 121.3	479	185.0 ± 94.9	0.001
Glucose	21	205.1 ± 71.4	138	175.3 ± 64.3	0.04
HbA1C	21	8.8 ± 2.5	138	7.7 ± 2.2	0.04
Inflammation markers					
CRP	61	77.1 ± 79.1	585	42.6 ± 56.5	<0.0001
FER	37	1,123.8 ± 971.6	428	462.4 ± 577.2	<0.0001
ESR	6	49.8 ± 35.6	42	29.9 ± 23.7	0.11

**Figure 2:**
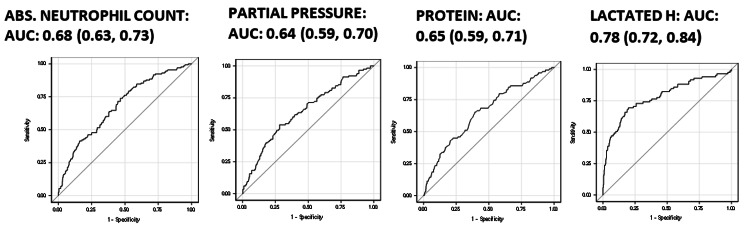
ROC curves depicting the power to discriminate those admitted to the ICU from those not ROC: receiver operating characteristic; AUC: area under the curve; ICU: intensive care unit; partial pressure: p50c (oxygen tension at which hemoglobin is 50% saturated)

The area under the curve (AUC) for all four predictors of ICU admission is above 0.50, and as shown in Figure 2, the curves do not cross the line of no discrimination at any point. The AUC (95% CI) is 0.68 (0.63, 0.73) for the absolute neutrophil count, 0.64 (0.59, 0.70) for partial pressure (p50c), 0.65 (0.59, 0.71) for protein, and 0.78 (0.72, 0.84) for LDH. With these four predictors included in a single model, the AUC (95% CI) is 0.76 (0.71, 0.80).

As shown in Table 5, the cut point to discriminate admissions to the ICU based on Youden’s J index is as follows: >4,200 for the absolute neutrophil count, >26.5 for partial pressure/p50c, <7 for protein, and >675 for LDH. A minimum value of 0.50 was ensured for sensitivity and specificity while determining the cut point.

The sensitivity and specificity of each of the four predictors are as follows: 0.75 (0.66, 0.82) and 0.51 (0.47, 0.56) for the absolute neutrophil count, 0.50 (0.41, 0.60) and 0.72 (0.67, 0.76) for partial pressure, 0.66 (0.56, 0.76) and 0.58 (0.54, 0.63) for protein, and 0.69 (0.58, 0.79) and 0.80 (0.76, 0.84) for LDH, respectively.

The positive likelihood ratio (LR+) of each of the four predictors is as follows: 1.54 (1.26, 1.85) for the absolute neutrophil count, 1.79 (1.25, 2.50) for partial pressure, 1.59 (1.21, 2.05) for protein, and 3.50 (2.43, 4.92) for LDH.

**Table 5:** Measures of validity of predictors of admission to the ICU ICU: intensive care unit;

ICU	Absolute neutrophil count	Partial pressure (p50c)	Protein	LDH
Cut point	>4,200	>26.5	<7	>675
Sensitivity	0.75 (0.66, 0.82)	0.50 (0.41, 0.60)	0.66 (0.56, 0.76)	0.69 (0.58, 0.79)
Specificity	0.51 (0.47, 0.56)	0.72 (0.67, 0.76)	0.58 (0.54, 0.63)	0.80 (0.76, 0.84)
LR+	1.54 (1.26, 1.85)	1.79 (1.25, 2.50)	1.59 (1.21, 2.05)	3.50 (2.43, 4.92)

As shown in Table 6, the allocation of points is as follows: 2 points each for the absolute neutrophil count, partial pressure/p50c, and protein and 7 points for LDH. For all other values, the points allotted are zero. When the total score is greater than 4 or more, the sensitivity and specificity is 0.70 (0.61, 0.77) and 0.70 (0.66, 0.73), respectively; the positive and negative predictive value is 0.34 (0.28, 0.39) and 0.91 (0.88, 0.94), respectively, and the positive likelihood ratio is 2.31 (1.80, 2.90). The risk of ICU admission is 5.3 (3.6, 8.0) times higher if the score is 4 or more.

**Table 6:** Allocation of points for predictors of ICU admission ICU: intensive care unit; OR: odds ratio; CI: confidence interval

Predictors	Cut point	OR (95% CI)	Adjusted OR (95% CI)	Score
Absolute neutrophil count	>4,200	3.1 (2.0, 4.8)	2.2 (1.2, 3.9)	2
Partial pressure (p50c)	>26.5	2.6 (1.7, 3.9)	2.2 (1.2, 4.0)	2
Protein	<7.0	2.8 (1.7, 4.4)	2.2 (1.2, 3.9)	2
Lactate dehydrogenase	>675	9.2 (5.4, 15.5)	7.1 (3.9, 12.9)	7

## Discussion

The COVID-19 pandemic put added pressure on the response capacity of public healthcare systems globally and more so in developing economies that suffer from pre-existing healthcare inequities [5]. Despite the advanced critical care interventions, critical COVID-19 admissions need prolonged ventilation and have high short-term mortality [6]. The sophistication and intensity of training required for the care of patients during pandemics place a manifold burden on healthcare delivery. A relatively small number of patients can easily overwhelm healthcare systems already overstressed by continuous demand for treatment of vector-borne diseases and high rates of non-communicable diseases [1].

This single-center retrospective review of our institutional database identifies potential predictors of mortality and the need for ICU care in COVID-19 who accessed healthcare at our academic setup. Anticipating multicollinearity as high due to how a physician orders blood tests, these naturally occurring groups were assessed using principal component analysis before including them in the final model. The decision to choose between the two lowest 1-R squared ratios as a cluster representative was made by a clinician (CCP) and a statistician (AD). Three predictors of mortality and four predictors of ICU admission were identified, of which only one predictor (absolute neutrophil count) was a common predictor of both ICU admission and mortality but with two separate cut points.

Predictors of mortality

Neutrophil Count

Our study found a strong association between absolute neutrophil counts and severity (as reflected by a need for ICU care) and mortality in COVID-19. An absolute neutrophil count of >4,200 predicted the need for ICU admission and >7,200 predicted mortality. This mirrors similar reports where neutrophil counts on the first day of hospitalization have been predictive of severity or who would later require transfer to the intensive care unit, preceding the onset of critical illness and predicting increased mortality [7-9]. A recent meta-analysis and regression also substantiate these observations [10]. The neutrophilia might be related to the cytokine storm typical of severe COVID-19 pneumonia and the associated poor prognosis [11].

Blood Urea

Higher urea and lower estimated glomerular filtration rate (eGFR) in ICU patients indicate the well-documented impact of COVID-19 on renal functions [12]. Increasing urea levels have been documented predictors of clinical worsening in COVID-19 [13]. We observed that a blood urea level greater than 45 was predictive of needing ICU care. The etiology of kidney disease involvement could be due to the virus entering kidney cells through an angiotensin-converting enzyme 2 (ACE2)-dependent pathway, direct cytopathic effects on kidney tissue, or deposition of immune complexes of viral antigen or virus-induced specific immunological effector mechanisms [13].

Ferritin

In our dataset, serum ferritin of >500 was predictive of ICU admission. Although ferritin is an iron-storing protein that helps diagnose iron deficiency anemia, it can also be a marker of viral replication as ferritin levels increase during viral infections. A similar increase has been reported in COVID-19 [14-16]. Serum ferritin is closely related to poor recovery in COVID-19 patients, and those with impaired lung lesions are more likely to have an increase in ferritin levels [17].

Predictors of ICU admission

p50c (p50 Is the Oxygen Tension at Which Hemoglobin Is 50% Saturated)

There are conflicting results relating to the oxygen dissociation curve in COVID-19 [18,19]. A higher p50 could correlate with better survival [20]. We observed a right shift of p50c in SARS-CoV-2 as a predictor of ICU care. In the injured lung, the saturation of hemoglobin is compromised, and in the tissues, associated anemia reduces the volume of delivered oxygen [21]. Although “happy hypoxia” is a reported feature in COVID-19 with preserved oxygen saturation despite low partial pressure of oxygen due to the leftward shift of the oxyhemoglobin dissociation curve induced by hypoxemia-driven hyperventilation as well as potential viral interactions with hemoglobin [22], the decreased affinity of hemoglobin for oxygen might likely be the compensatory mechanism in patients with increasing COVID severity to improve the deposition of oxygen in tissues [23].

Serum Protein

COVID-19 is associated with a hypercatabolic state that entails excessive protein loss. Hypoproteinemia has been reported as a marker of COVID-19-related inflammatory exacerbation and disease progression [24]. In our analysis, a serum protein of less than 7 g/dL (OR: 2.8 (95% CI: 1.7, 4.4)) was a predictive variable for ICU admission. COVID-19 inflammation could potentiate glycation stress, forming toxic metabolites that accelerate inflammation and oxidative stress, leading to cellular protein damage [25]. Compounding this could be the inadequate food supply secondary to symptoms such as dysgeusia, anorexia/vomiting, and diarrhea [26].

Lactate Dehydrogenase (LDH)

An LDH value of >675 was predictive of severity with the need for ICU admission in our series. Similar observations in other case series have noted an elevation in LDH as predictive of clinical worsening [27]. A meta-analysis aimed to evaluate the prognostic performance of elevated lactate dehydrogenase (LDH) in patients with COVID-19 showed that an increase would indicate a 44% posterior probability, and non-elevated LDH would indicate an 11% posterior probability for poor prognosis [28]. A recent study identified increasing LDH with the need for oxygen requirements in COVID-19 [29]. Although used as a marker of cardiac damage, abnormalities in LDH values can also result from injuries to other organs and are also reflective of decreased oxygenation and upregulation of the glycolytic pathway [27]. In severe COVID-19 infections (as a severe form of interstitial pneumonia), more LDH could be released in circulation since LDH is present in lung tissues [30].

Predictive scores

We assigned a score to each predictive variable based on the adjusted odds ratio (4 points for absolute neutrophil count, 8 points for urea, and 3 points for ferritin). When the total score was 7 or more, the mortality risk increased to 10.5 (6.3, 17.5) times higher. When the total score was 7 or more, the sensitivity and specificity were 0.63 (0.51, 0.74) and 0.86 (0.83, 0.89), respectively.

A similar score for ICU admission was derived (2 points each for absolute neutrophil count, partial pressure/p50c, and protein and 7 points for LDH). When the total score was 4 or more, the sensitivity and specificity were 0.70 (0.61, 0.77) and 0.70 (0.66, 0.73), respectively.

Limitations

There are several limitations to this work. There is likely to be significant heterogeneity between different hospitals regarding diagnostic facilities available and access to the ICU and supportive care. Our prediction model was validated only in the development cohort of 757 patients. It was not validated again in a separate cohort. We included the most known predictive parameters of worse outcomes in patients with COVID-19 at the time of the inclusion of our study population. However, we could not incorporate all known clinical parameters and laboratory values such as body mass index, history of tobacco use, CT score, and pulmonary function tests mainly because they were not available for all patients.

## Conclusions

In conclusion, we identified a set of predictive variables to recognize the subset of patients with the highest risk of death or clinical worsening requiring ICU care. It is our opinion that this set of variables, which include neutrophil count, blood urea, ferritin, p50c, serum protein, and lactate dehydrogenase (LDH), are readily available and accessible to most centers admitting patients with COVID-19 irrespective of the resource. The markers relate to the recognized and hypothesized biological impact of COVID-19 in inflammation, coagulation, oxygenation, and metabolism domains. The scores are informative in prognosticating and could enable more clarity in clinician and provider discussions, making it useful for triaging patients to the limited number of intensive care or high-dependency unit beds. Validating these scores prospectively to predict ICU care or death in other cohorts will establish the usefulness of this scoring system. Clinicians should pay close attention to these markers and intervene early.
